# Perinatal exposure to tetracycline contributes to lasting developmental effects on offspring

**DOI:** 10.1186/s42523-021-00099-z

**Published:** 2021-05-11

**Authors:** Elizabeth M. Hill, Christopher D. Howard, Tracy L. Bale, Eldin Jašarević

**Affiliations:** 1 Center for Epigenetics Research in Child Health and Brain Development, Department of Pharmacology, University of Maryland School of Medicine, Baltimore, USA; 2 Department of Pharmacology, University of Maryland School of Medicine, Baltimore, MD USA

**Keywords:** Microbial assembly, Behavior, Tetracycline inducible expression, Tet-On/Off System, Development

## Abstract

**Background:**

For more than 30 years, the tetracycline on/off system of inducible gene expression has been leveraged to study disease mechanisms across many research areas, especially that of metabolism and neuroscience. This system requires acute or chronic exposure to tetracycline derivatives, such as doxycycline, to manipulate gene expression in a temporal and tissue-specific manner, with exposure often being restricted to gestational and early developmental windows. Despite evidence showing that early life antibiotic exposure has adverse effects on gut microbiota, metabolism, physiology, immunity and behavior, little is known regarding the lasting impact of doxycycline treatment on relevant outcomes in experimental offspring.

**Results:**

To examine the hypothesis that early life doxycycline exposure produces effects on offspring growth, behavior, and gut microbiota, we employed the most commonly used method for tetracycline on/off system by administering a low dose of doxycycline (0.5 mg/ml) in the drinking water to C57Bl/6J and C57BL/6J:129S1/SvImJ dams from embryonic day 15.5 to postnatal day 28. Developmental exposure to low dose doxycycline resulted in significant alterations to growth trajectories and body weight in both strains, which persisted beyond cessation of doxycycline exposure. Developmental doxycycline exposure influenced offspring bacterial community assembly in a temporal and sex-specific manner. Further, gut microbiota composition failed to recover by adulthood, suggesting a lasting imprint of developmental antibiotic exposure.

**Conclusions:**

Our results demonstrated that early life doxycycline exposure shifts the homeostatic baseline of prior exposed animals that may subsequently impact responses to experimental manipulations. These results highlight the gut microbiota as an important factor to consider in systems requiring methods of chronic antibiotic administration during pregnancy and critical periods of postnatal development.

**Supplementary Information:**

The online version contains supplementary material available at 10.1186/s42523-021-00099-z.

## Background

The tetracycline on/off system of inducible gene expression is a commonly used genetic tools that enabled precise control of gene expression in a reversible, temporal, and tissue-specific manner [1–3]. This system regulates the activation or repression of genes through a bacterial construct that is controlled by the addition or withdrawal of tetracyclines, typically doxycycline, and frequently requiring exposure to these antibiotics during pregnancy and critical periods of development [4–12]. Adverse effects of administering 1 mg/ml doxycycline in drinking water during pregnancy and early life development were reported in the earliest studies utilizing tetracycline inducible expression, including severe cognitive impairments and teratogenic defects in exposed offspring, leading to more recent studies using this system to reduce the doxycycline dose by half (0.5 mg/ml) [9, 13, 14]. However, the potential adverse effects of perinatal exposure to even this low dose of doxycycline on relevant outcomes in experimental offspring has not been evaluated.

Administration of antibiotics during the pregnancy and the postnatal period adversely impacts many aspects of offspring development, most notably the assembly and maturation of the gut microbiota[15–17]. Even brief exposures to antibiotics during this critical window of development has been associated with lasting consequences on gut microbiota, metabolism, immunity, and behavioral outcomes[16, 18, 19]. Despite the mounting evidence suggesting that antibiotic treatment early in life disrupts the gut microbiota and influences weight gain, many studies utilizing tetracycline inducible expression have not included an antibiotic-only wild-type control to reconcile the effects of antibiotic-mediated disruption of the microbiota on physiology and behavior. Thus, to determine whether doxycycline exposure during pregnancy and the early postnatal period influences offspring outcomes independent of experimental manipulation, we exposed pregnant C57Bl/6J and C57BL/6J:129S1/SvImJ mice to 0.5 mg/ml doxycycline with 0.2 % sucrose (Dox) or sucrose-only (Control) from embryonic day 15.5 through postnatal day 28. Resulting offspring were assessed for phenotypes commonly examined in studies utilizing tetracycline inducible expression, including body weight, HPA stress axis responsivity, and general activity patterns. Given the importance of the gut microbiome to physiological health, longitudinal analysis of the gut microbiota with 16 S ribosomal RNA marker gene sequencing from postnatal day 21 to 63 was used to examine the impact of doxycycline on the assembly of the gut microbiota during development and subsequent recovery following the cessation of antibiotic exposure.

## Results

### Developmental exposure to doxycycline exerts lasting effects on offspring body weight and gut morphology

**Fig. 1:**
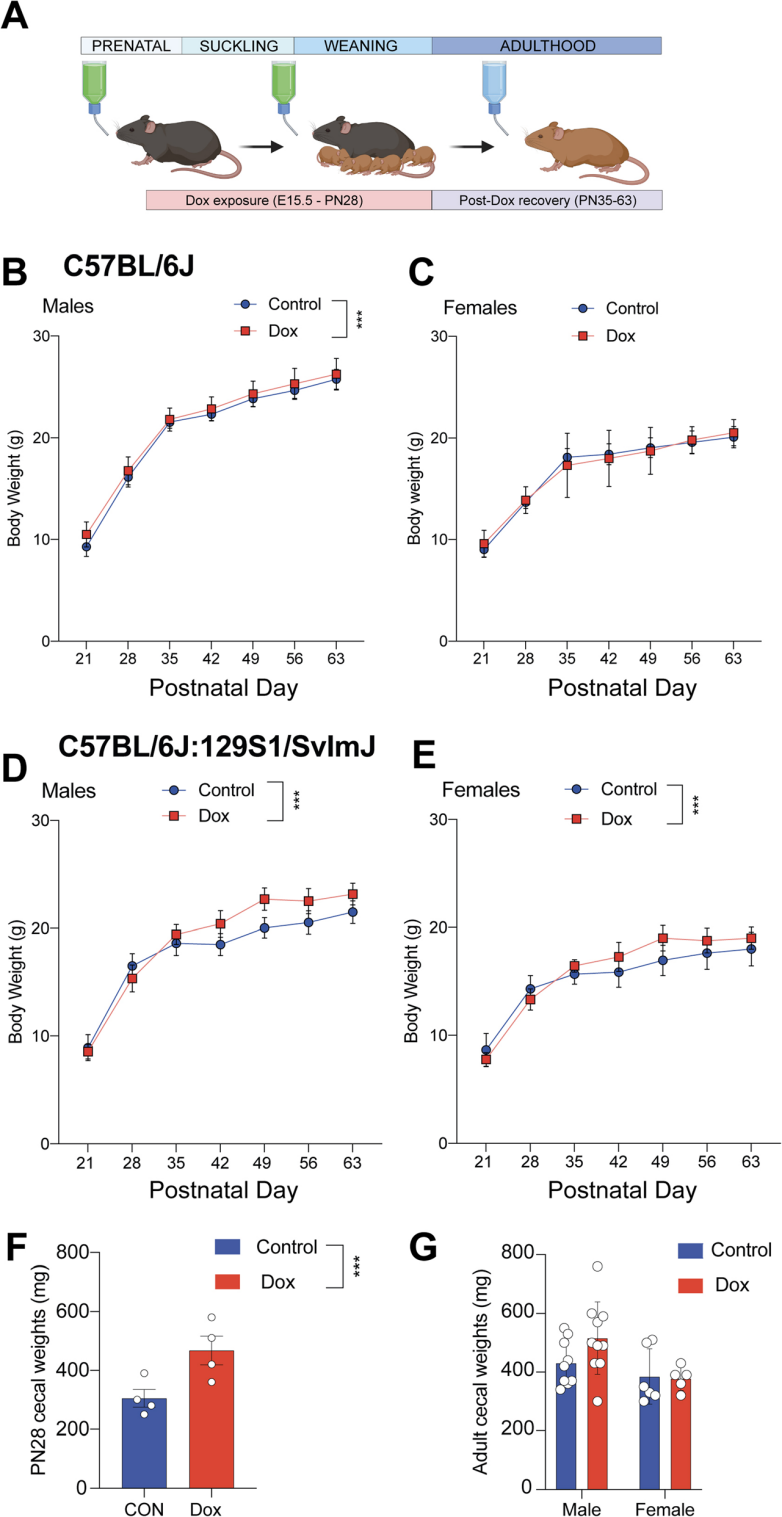
Developmental exposure to doxycycline exerts lasting effects on offspring body weight and gut morphology. **a** Experimental timeline to assess the lasting effects of developmental exposure to doxycycline on offspring outcomes. **b** C57Bl/6J male offspring body was changed significantly over time (two-way ANOVA, main effect of time, F_6, 175_ = 557.3, P < 0.0001.) and between treatment groups (two-way ANOVA, main effect of treatment, F_1, 175_ = 11.15, P= 0.001). **c** C57Bl/6J female offspring body weight was significantly changed over time (two-way ANOVA, main effect of time, F_6,132_= 606.0, P<0.0001), but not between treatment groups (two-way ANOVA, main effect of treatment, F_1,22_= 0.0002955, P=0.9864). **d** Body weight experiments with animals of a mixed C57BL/6J:129S1/SvImJ background, male offspring body weight was significantly changed over time (two-way ANOVA, main effect of time, F_6, 186_= 1191, P < 0.0001) and across treatment (two-way ANOVA, main effect of treatment, F_1, 31_= 14.57, P = 0.0006). **e** In the mixed background experiment, female offspring body weight was significantly changed across time (two-way ANOVA, mean effect of time, F _1, 127_ = 204.5, P < 0.0001) and across treatment (two-way ANOVA, main effect of treatment, F_6,127_= 10.50, P =0.0017). **f** Following cessation of doxycycline exposure, the cecal weights of mixed background males and females were greater compared with control males and females at postnatal day 28 (t_6_= 2.844, P = 0.0294). **g** This effect was resolved by postnatal day 63 Male (t_17_ = 1.758, P = 0.0967) Female (t_9_ = 0.1532, P = 0.8816). Data represented as mean ± SD. * *P *< 0.05, ** *P *< 0.01, ***P < 0.001. C57 body weight experiment: Control male n = 9; Dox male n = 18; Control female n = 14; Dox female n = 10. Mixed background body weight experiment: Control Male n= 13; control female n= 8; Dox male n = 20; Dox female n = 11. Cecal Weight experiments: postnatal day 28 n = 4; postnatal day 70-90, male n = 9-10; female = 5

Tetracycline inducible expression requires the administration of doxycycline to activate or silence targeted genes. Given the established effects of antibiotic exposure on growth, metabolism, and behavior, we assessed the specific effect of developmental low-dose doxycycline exposure on phenotypes by measuring body weight, cecal weight, and behavior of offspring across treatment groups [15, 20]. To determine whether developmental exposure to doxycycline affects offspring body weight across puberty and into adulthood, weekly body weights were monitored from postnatal day 21 to 63 across treatment groups. The body weight curve of doxycycline (Dox) C57Bl/6J males was significantly increased in comparison to control males (P = 0.001) (Fig. 1b). In female C57Bl/6J mice, however, doxycycline exposure did not significantly change the body weight curve across the lifespan (P = 0.9864) (Fig. 1 c).

To examine whether host genetics are a significant factor in determining sex-specific body weight gain following doxycycline exposure, mixed colony C57Bl/6J:129S1/SvImJ (mixed background) mice were exposed to doxycycline as described above, and weekly body weights were compared across treatment groups from postnatal day 21 to 63. Replication with mixed background animals resulted in different weight phenotypes than a purely C57Bl/6J background. Consistently with our prediction, mixed colony Dox males showed excessive weight gain compared with control males (P = 0.0006) (Fig. 1d). In addition, Dox females showed a significant difference in body weight curve from control females (P = 0.0017) (Fig. 1e). To determine whether doxycycline exposure impacts gastrointestinal morphology, cecal samples on postnatal day 28 offspring were collected across treatment groups (Fig. 1 f). Cecal weight on postnatal day 28 was increased in Dox offspring compared with control offspring (P = 0.0294). Cecal weights on postnatal day 70 were also taken from a subset of males and females from control and Dox groups. In adulthood, cecal weights between Dox and control animals were not different in males (P = 0.0967) or females (P = 0.8816) (Fig. 1g).

**Fig. 2:**
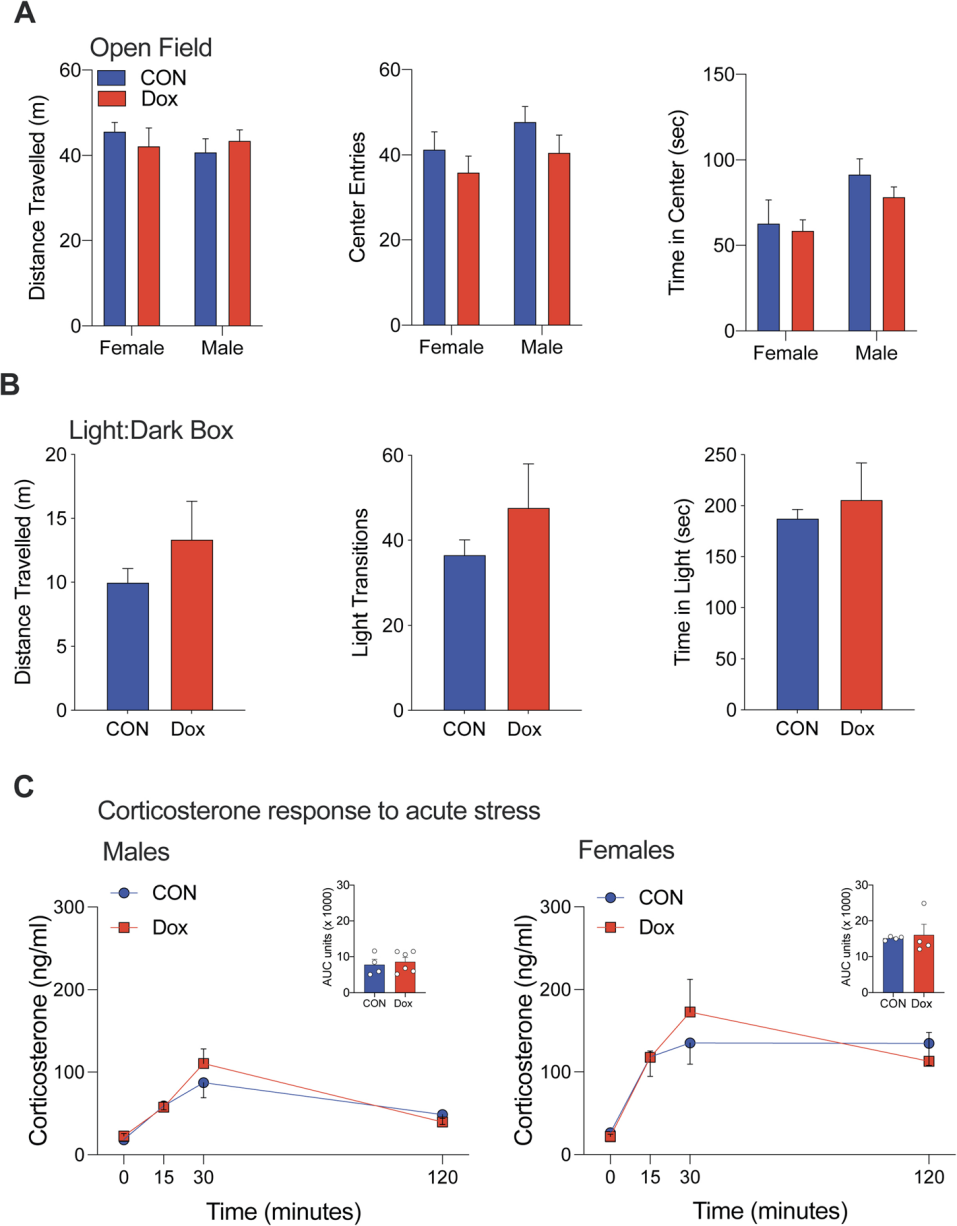
Motor function, locomotion and response to acute stressors is intact of males and females exposed to doxycycline during development. **a**) No effect of developmental exposure to doxycycline on distance traveled, center entries or time in center in the open field in adult males and females. **b** No effect of developmental exposure to doxycycline on distance traveled, center entries or time in center in the light: dark box in a cohort of adult males. **c** No effect of developmental exposure to doxycycline on the corticosterone response to an acute restraint stress in adult males and females. 4-6 animals per sex per treatment groups were used for these studies, all *p*s > 0.05

To determine whether male-specific differences in body weight were associated with changes in motor function, locomotion, and corticosterone response to an acute stressor, we examined outcomes in adult control and Dox male offspring in the open field, light-dark box, and measured circulating corticosterone to an acute restraint stress. No differences were observed between Dox and control animals across this battery of tests, suggesting that developmental exposure to low-dose Dox did not significantly impair locomotion, motor function, or corticosterone response to an acute stressor (Fig. 2).

### Doxycycline disruption of offspring metabolism is associated with lasting alterations to the offspring gut microbiota

**Fig. 3:**
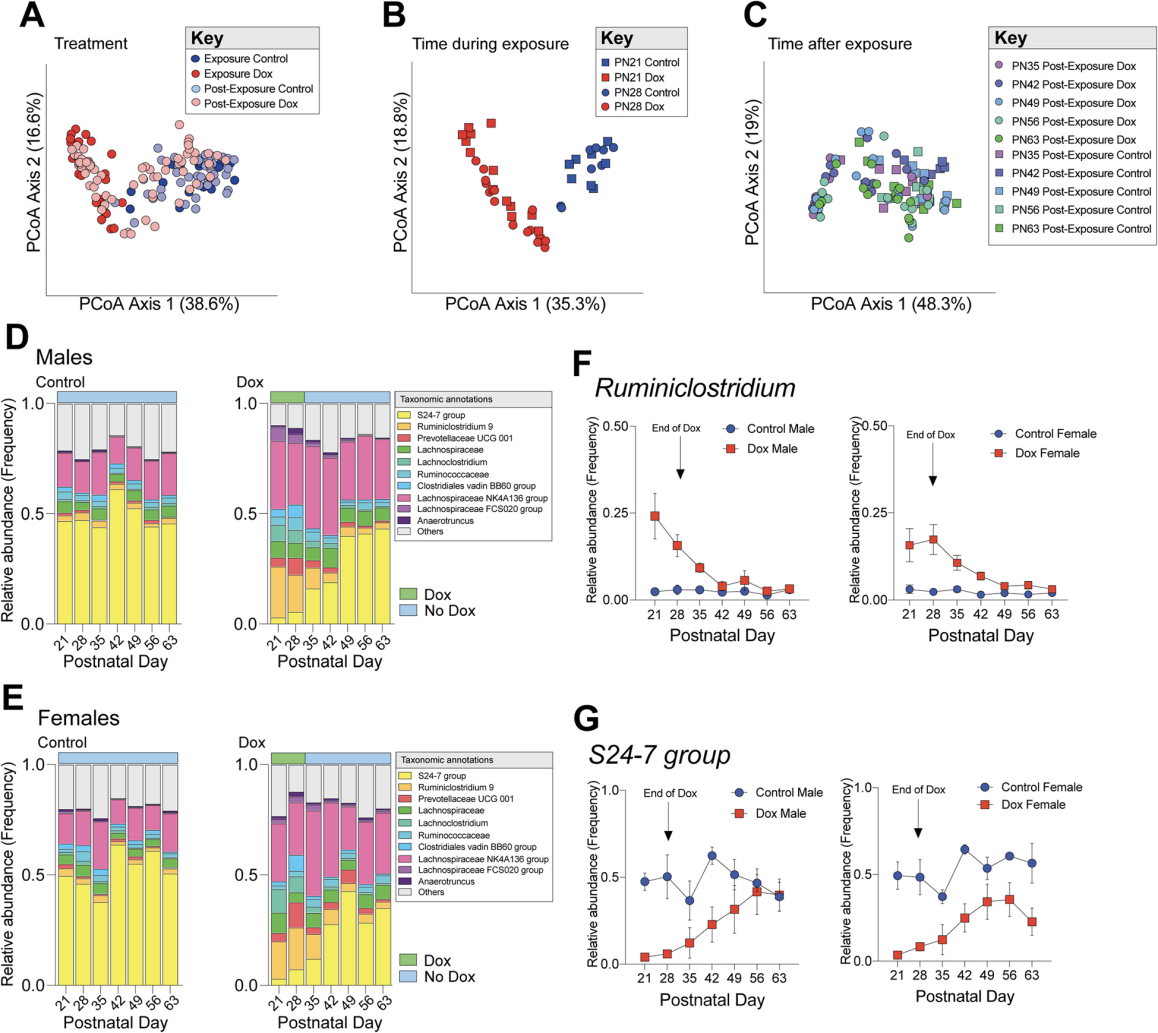
Doxycycline disruption of offspring metabolism is associated with lasting alterations to the offspring gut microbiota. **a** Principal coordinates analysis comparing fecal microbial community structure in male and female offspring from Control and Dox groups throughout the duration of collection postnatal day 21 to 63. Doxycycline treatment significantly drives community structure. (PERMANOVA, F = 42.772, r^2^ = 0.22304, P < 0.001). **b** Principal coordinate analysis comparing fecal microbial community structure in Dox offspring compared to controls during doxycycline exposure postnatal day 21 to 28. Doxycycline significantly drives community structure in offspring (PERMANOVA, F = 27.176, r^2^ = 0.39286, P < 0.001). **c** Principal coordinate analysis comparing fecal microbial community structure of Dox offspring compared to controls after exposure to doxycycline ends. Doxycycline still drives community structure in a number of Dox samples, however, communities shift to become more like Controls as they reach postnatal day 63 (PERMANOVA, F = 26.046, r^2^ = 0.19876, P < 0.001). **d** Stacked bar plots show the average relative abundance of the ten most-abundant taxa in Dox and Control male offspring during doxycycline exposure at postnatal day 21 and 28 and post-DOX exposure at postnatal day 35 to 63. Dox exposed offspring show major differences in community composition particularly a loss of Bacteroidetes and a simultaneous bloom of Firmicutes. **e** Stacked bar plots show the average relative abundance of the ten most-abundant taxa in Dox and Control female offspring during doxycycline exposure at postnatal day 21 and 28 and post-DOX exposure at postnatal day 35 to 63. **f** *Ruminiclostridium* is significantly changed in males over time (two-way ANOVA, main effect of time, F_6, 51_ = 4.1063, P = 0.0066), by doxycycline treatment (two-way ANOVA, main effect of treatment, F_1, 9_ = 11.6871, P = 0.0076), and their interaction (two-way ANOVA, time x treatment interaction, F_6, 51_ = 3.8514, P < 0.05). In females, *Ruminiclostridium* was significantly changed over time (two-way ANOVA, main effect of time, F_6, 54_ = 3.471, P = 0.0057), by treatment (two-way ANOVA, main effect of treatment, F_1, 9_ = 13.78, P = 0.0048) and their interaction (two-way ANOVA, time x treatment interaction, F_6, 54_ = 2.578, P = 0.0287). Post hoc analysis revealed that bloom of Ruminiclostridium during active Dox exposure was reduced after exposure ended. Male: (Sidak’s multiple comparisons, t_60_ = 1.594, P = 0.6344), Female: (Sidak’s multiple comparisons, t_63_ = 2.115, P = 0.2398). **g** *S24-7* group shows temporal patterns during doxycycline exposure and post-exposure. In males, abundance of *S24-7* was significantly changed by treatment (two-way ANOVA, main effect of treatment, F_1,9_ = 12.3622, P = 0.0066). Post hoc analysis revealed that this taxon increased to control levels by postnatal day 63 (Sidak’s multiple comparisons, t_60_ = 0.0641, P > 0.999). *S24-7* in females was significantly changed over time (two-way ANOVA, main effect of time, F_6, 54_ = 3.1799, P = 0.0096) and by treatment (two-way ANOVA, main effect of treatment, F_1, 9_ = 33.3202, P = 0.0003) with incomplete recovery at postnatal day 63 (Sidak’s multiple comparisons, t_63_ = 2.9151, P = 0.0339). Relative abundance for F and G is reported as mean frequency ± SEM. 4–7 samples per sex, treatment, and timepoint were used for analyses

Given the well-established link between the microbiota, gut function and body weight, we next determined whether developmental doxycycline exposure disrupted assembly of the offspring gut microbiota. Weekly fecal samples were collected from offspring across treatment groups from postnatal day 21 to 63 and analyzed using microbiome profiling by targeted sequencing of the V4 region of the 16 S rRNA gene. To determine whether doxycycline altered the assembly of the gut microbiome of offspring across important maturation periods, including weaning, puberty and adulthood, beta-diversity was calculated and compared as a function of treatment “Exposure” (postnatal day 21–28) vs. “Post-Exposure” (postnatal day 35–63). Bray-Curtis divergence matrices were calculated to determine the distances between control and Dox offspring during Exposure and Post-Exposure and then visualized using principal coordinate analysis (Fig. 3 a). Permutational multivariate analysis of variance (PERMANOVA) showed significant differences in microbial community structure between Control and Dox animals (p < 0.001). Further, no sex differences in microbiota community structure were observed in Dox animals, suggesting that early-life antibiotic administration may decrease the magnitude of sex differences in gut microbiota.

We next determined temporal effects of antibiotic-mediated microbiota alterations during active doxycycline consumption and exposure. Beta-diversity analyses were repeated across treatment groups for postnatal day 21 and 28, the window of direct doxycycline exposure (Fig. 3b). Analysis by PCoA confirmed a main effect of doxycycline treatment of gut microbiota community structure (p < 0.001). Consistent with potency effects of doxycycline on the gut microbiota, no main effects of sex and time on community structure were detected. Differential abundance analysis revealed 37 differentially abundant taxa between control and Dox offspring during doxycycline exposure (Supplemental Table 1). The *Bacteroidales S24-7* genus was the dominant taxa in male and female offspring between postnatal days 21 and 28. Both male and female Dox offspring showed a drastic reduction in this taxon, with a concomitant bloom of Firmicutes including *Ruminiclostridium*, *Lachnospiraceae*, and *Lachnoclostridium*. In addition to the expansion of Firmicutes, Dox offspring showed significant increases in Proteobacteria such as *Escherichia/Shigella* and *Helicobacter* and other taxa such as *Rikenella*, *Prevotellaceae*, and *Desulfovibro* (all FDR < 0.05). Doxycycline treatment significantly decreased taxa such as *Candidatus Arthomitus*, *Oscillibacter*, and *Peptococcaceae* (all FDR < 0.05). Taken together, these results demonstrated that doxycycline exposure exerts significant effects on microbiota composition during a critical weaning period.

To determine temporal microbial dynamics following the withdrawal of doxycycline exposure at postnatal day 28, post-exposure fecal samples were collected from postnatal day 35 to 63 across treatment groups. Additional PCoA visualization of beta-diversity revealed that doxycycline treatment significantly drove community structure from postnatal day 35–63 after exposure had ended (Fig. 3 c) (p < 0.001). At postnatal day 35, a week after doxycycline exposure ended, both Dox males and females maintained the reduction of *Bacteroidales S24-7* and dominance of Firmicutes like *Lachnospiraceae* (Fig. 3e). At this time point, 31 taxa were significantly differentially abundant between control and Dox samples, 19 in males and 12 in females, indicating persisting effects of doxycycline a week after exposure ends (Supplemental Table 2a,b). From postnatal day 42 to 63, the major taxa that dominate the Dox microbiota shifted to resemble controls. At postnatal day 63, however, 14 taxa remained differentially abundant, 3 in males and 11 in females, indicating that despite major taxa rebounding some effects of doxycycline remain (Supplemental Table 3a,b). Of the microbiota changed by Dox, *S24-7* and *Ruminiclostridium 9* showed distinct temporal patterning. *Ruminiclostridium 9* significantly changed in males over time (p = 0.0066) and by doxycycline treatment (P = 0.0076). In females, *Ruminiclostridium 9* significantly changed over time (p = 0.0057), by treatment (p = 0.0048) and by their interaction (p = 0.0287). Post hoc analysis revealed that bloom of *Ruminiclostridium* during active Dox exposure was reduced after exposure ended (P = 0.6344; Females: P = 0.2398) (Fig. 3 f). In males, *S24-7* was significantly reduced by doxycycline treatment (p = 0.0066) and rebounded after postnatal day 42. Post hoc analysis revealed that this taxon increased to abundance of control males by postnatal day 63 (p > 0.999) (Fig. 3g). The family *S24-7* in Dox females was significantly changed over time (p = 0.0096) and by treatment (p = 0.0003), and Dox females showed an incomplete recovery of the taxon with differences persisting at postnatal day 63 (p = 0.0339). Collectively, these results demonstrated that doxycycline exposure exerted lasting effects on the gut microbiota during puberty and into adulthood, well beyond direct antibiotic exposure.

## Discussion

The tetracycline inducible system has been widely adopted to manipulate genes in a tissue-specific and temporal manner, particularly in research areas focused on metabolism, brain function and behavior. Temporal control of gene expression has made this system a valuable tool. Early studies that utilized the system reported that administration of doxycycline at 1 mg/ml in drinking water during development have been shown to exhibit teratogenic effects that is independent of genetic manipulation[9]. More recent applications have lowered doxycycline dose to 0.5 mg/ml in drinking water to prevent the teratogenic effects associated with antibiotic exposure[21–23]. However, the possibility that exposure to this lower dose during development influences the phenotypic outcomes independent of tetracycline inducible expression has not been examined. To determine whether low dose doxycycline exposure impacts offspring outcomes through disruption of the gut microbiota, we exposed pregnant dams to 0.5 mg/ml doxycycline and 0.2 % sucrose from embryonic day 15.5 to postnatal day 28. Given the history of antibiotics as growth promoters, we determined whether gestational and early developmental doxycycline exposure impacts offspring body weight into adulthood by monitoring weekly body weights from postnatal day 21 to 63[24]. Developmental doxycycline exposure significantly increased the body weight curve of C57Bl/6J males compared to control males. Dox C57Bl/6J females did not show any significant difference in body weight through adulthood despite showing similar shifts in the microbiome compared to Dox-exposed C57Bl/6J males. Several studies have shown that female mice are resistant to weight gain in response to dietary challenges, and may suggest important interactions between antibiotic exposure during development, the microbiome, sex differences, and host genetics[25–27]. The mechanisms controlling resistance to weight gain in C57Bl/6J females exposed to doxycycline remain unclear and could be explored in future studies.

Baseline strain-specific differences in genetics, metabolism, and behavior may influence the response to antibiotic exposure[28–31]. To determine whether the developmental effects of doxycycline exposure are specific only to the C57Bl/6 strain, we used the same experimental design in a mixed C57Bl/6J:129S1/SvImJ strain. Similar to C57Bl/6J offspring, doxycycline exposure contributed to an increase of offspring body weight in adulthood. Further, mixed background animals showed a greater magnitude of response as both Dox males and females were significantly heavier than controls. Both pure 129 and mixed C57Bl/6J:129S1/SvImJ strains have been shown to be more responsive to dietary challenges than purely C57Bl/6J strains, confirming strain-specific effects of antibiotic exposure on weight gain responses[32]. A possible explanation for strain-specific differences in the magnitude of body weight gain following developmental exposure to doxycycline is that the mouse strains were reared in different animal husbandry facilities, a known environmental contributor to variation in gut microbiota and body weight in rodents[33–37]. While animals were reared in similar environments for these experiments, including diet, bedding and methods of water treatment, the possibility remains that additional unexplored factors may explain our observed differences in gut microbiota and body weight between the two strains. Nevertheless, these results underline the importance of considering strain differences when planning research models, especially those involving doxycycline, body weight phenotypes, or developmental timepoints.

Antibiotic-mediated disruption of the gut microbiota has been linked to broad effects on weight gain, obesity, and metabolic dysfunction[16, 38, 39]. To determine whether developmental exposure to doxycycline influences the assembly of the gut microbiome and whether these changes could be associated with weight gain, we collected weekly fecal samples from offspring across treatment groups from postnatal day 21 to 63 and performed 16 S rRNA marker gene sequencing. Doxycycline significantly drove microbial community structure in both males and female offspring during exposure and after exposure ceased. This indicated that the developmental exposure of doxycycline was disruptive to the normal assembly of the microbiome. Differential abundance analysis revealed that Dox offspring showed sex-specific reduction in the family S24-7, a dominant taxa that colonizes the murine intestinal tract. Reduction in the relative abundance of this dominant taxon may alter the microbial ecology of the intestinal tract such that other microbiota may increase in abundance as a response. Consistent with this notion, we observed that reduction of S24-7 abundance was associated with the increased abundance of several taxa including *Escherichia/Shigella*. A bloom of Proteobacteria in the gut has been implicated in weight gain and inflammation, and an increase of this taxa early in life may contribute to the weight gain that was observed in the C57Bl/6J male[40, 41]. Differential abundance analysis also revealed that the number of differentially abundant taxa between Dox and control groups was gradually reduced with each timepoint following cessation of doxycycline exposure, suggesting that the microbiota underwent significant community remodeling following the withdrawal of antibiotic treatment. However, compositional differences remained in adult Dox offspring, suggesting that the incomplete recovery from developmental antibiotic exposure may reflect lasting alterations to the host but this hypothesis will require direct testing. Our results add to previous work showing that recovery of microbial communities following antibiotics is protracted process that is influenced by complex interactions between sex and age[42, 43]. Given that the assembly of the gut microbiota during development is critical for the maintenance of growth, development, and proper immune function, our results point to additional implications regarding developmental doxycycline administration on the functioning of the immune system of experimental offspring that should be the focus of future investigations[44, 45] .

## Conclusions

Our results suggest that early life exposure to a standard low-dose of doxycycline used in tetracycline inducible expression studies contributes to body weight differences and alterations to the gut microbiota during critical periods of weaning and puberty. These results suggest that antibiotic administration during the developmental period is able to shift the homeostatic baseline of exposed animals in a manner that may impact response to subsequent manipulations. Studies that rely on body weight as a physiological readout for normal growth and development are most vulnerable to confounding effects of doxycycline exposure. Further, these results further highlight the gut microbiota as an important confound to consider in systems that require antibiotic exposure during pregnancy and critical periods of postnatal development.

## Methods

### Experimental animals

Mice used for weight studies were C57Bl/6J purchased from The Jackson Laboratory and arrived at the University of Pennsylvania at 4 weeks of age. These animals were then left to acclimate to the conditions present within the University of Pennsylvania’s Hill Pavilion vivarium. C57Bl/6J breeding pairs (n = 11; 5 Control and 6 Doxycycline) were used for these studies, and females were checked daily at 0700 EST for copulation plugs. Dams were housed under a 12 h light/ day photoperiod with a standard chow diet (Purina Rodent Chow, St. Louis, MO; 28.1 % protein, 59.8 % carbohydrate, 12.1 % fat) and ad libitum access to water. Embryonic day 0.5 (E0.5) was determined to be at 1200 EST of the day the copulation plug was observed. All experiments were approved by the University of Pennsylvania Institutional Animal Care and Use Committee and performed in accordance with National Institutes of Health Animal Care and Use Guidelines. Body weight experiments were repeated with a mixed background strain (C57BL/6J:129S1/SvImJ) and were performed at the University of Maryland School of Medicine Health Sciences Facility III’s vivarium. A total of 8 C57Bl/6J:129S1/SvImJ breeding pairs (n = 8; 4 Control and 4 Doxycycline treated) were used for these studies. All analysis was conducted at the level of the litter to control for potential litter and cage confounds. All experiments were approved by the University of Maryland School of Medicine Institutional Animals Care and Use Committee Guidelines.

### Doxycycline-containing water

Commercially available doxycycline hyclate (catalog # D9891, Sigma, St. Louis, MO) and sucrose (catalog # S9378, Sigma, St. Louis, MO) were used to prepare 500-mL drinking solutions. Doxycycline-treated animals were provided ad libitum access to 500-mL drinking solutions containing 0.5 mg/ml doxycycline hyclate and 0.2 % sucrose, whereas control animals were provided ad libitum access to 500-mL of a 0.2 % sucrose solution. Pregnant dams were given doxycycline treatment from embryonic day 15.5 until postnatal day 28. Due to its light sensitive properties and reduced stability at room temperature, water feeding bottles containing doxycycline hyclate were wrapped in aluminum foil with water feeding bottles being replaced with fresh solution every 3 days. When offspring were weaned from dams at postnatal day 28, both Control and Dox offspring were transferred to cages with untreated water.

### Characterization of physiological and behavior phenotypes

Same-sex littermates were split into two groups for subsequent physiological and behavioral characterization (1 male and 1 female per litter per group). Groups were examined for HPA stress axis responsiveness, performance in the light-dark (LD) box and performance in the open field test. A minimum 7 d recovery period separated tests and ordering of tests were counter-balanced.

### Hypothalamic-pituitary-adrenal stress axis responsiveness

Testing occurred on 10-week-old control and doxycycline-exposed mice 2 h after lights on (0900 EST). Mice were restrained in animal restrainers for 15 min beginning at time 0 and were immediately returned to their home cage at the conclusion of restraint. Plasma was collected by obtaining blood from a single distal tail snip (< 1mm tissue) at 0 min, which sufficed for repeated blood collections at 15-, 30- and 120-min time points. A total 10 uL of tail blood was collected at each time point by micropipette into EDTA-treated tubes, first blotting the blood clot and then gently milking the tail to stimulate blood flow. Tail blood collection required < 1 min to complete and samples were immediately placed on ice. Blood samples were then centrifuged for 10 min at 5000 rpm. Plasma was collected and stored at − 80 °C until analysis. Corticosterone levels were determined by 125I-corticosterone radioimmunoassay (MP Biomedicals, Orangeburg, NY) using 3 µL of plasma as per kit instructions. The minimum detection limit of the assay was 7.7 ng/ml, and the intraassay coefficient of variation was 7.1 %.

### Light-dark box

Mice were exposed to the light-dark box to determine the effect of doxycycline exposure to anxiety-like behavior. The light-dark box test was performed as previously described (Bale et al., 2000). Adult mice (n = 6–8 per group) were placed in the light compartment at the beginning of the 10-min test session. Light intensities were set at 5 lx in the dark compartment and 300 lx in the light compartment. All testing occurred 2–5 h after lights off. Total time spent in the light compartment and the number of light-to-dark transitions were analyzed with ANY-maze v4.75 software (Stoelting Co., Wood Dale, IL). Mice were scored by an investigator blind to the treatment group.

### Open field

Mice were exposed to the open field test during the dark cycle 1 week after completion of the light-dark box test to determine the effect of doxycycline exposure on anxiety-like behavior and locomotor activity. The open field apparatus consisted of a white Plexiglass box 50 × 50 × 22 cm with (16) 12 × 12 cm squares drawn on the floor. Testing was conducted with 120 lx in the center of the box, 2– 4 h after lights out (9:00 to 11:00 P.M.). Each mouse was placed in the center of the box to initiate the 5 min test. Mice were scored for total line crossings and center crosses by an investigator blind to the treatment group.

### Fecal bacterial DNA isolation

Weekly fecal samples were collected from 1 male and 1 female per litter per group from C57BL/6J cohort from postnatal day 21 to 63. Genomic DNA from fecal samples were isolated using the Stratec PSP Spin Stool DNA Plus kit as per the manufacturer’s protocol for “difficult-to-lyse bacteria” (STRATEC Molecular GmbH, Berlin, Germany).

### Illumina MiSeq 16 S rRNA marker gene sequence data processing and analysis

The V4 region of the bacterial 16 S rRNA gene was amplified using a dual-index paired-end sequencing strategy for the Illumina platform as previously described. Sequencing was performed on a MiSeq instrument (Illumina, San Diego, CA) using 2 × 250 base paired-end chemistry at the University of Maryland School of Medicine Institute for Genome Sciences. The sequences were demultiplexed using the dual-barcode strategy, a mapping file linking barcode to samples and split_libraries.py, a QIIME-dependent script. The resulting forward and reverse fastq files were split by sample using the QIIME-dependent script split_sequence_file_on_sample_ids.py, and primer sequences were removed using TagCleaner (version 0.16). Further processing followed the DADA2 workflow for Big Data and DADA2 (v.1.5.2) (https://benjjneb.github.io/dada2/bigdata.html). The sequencing run yielded a total of 20,712,200 reads with an average read count of 11,972. Data filtering was set to include features where 10 % of its values contain a minimum of 4 counts. Data filtering was set to include features where 10 % of its values contain a minimum of 4 counts. In addition, features that exhibit low variance across treatment conditions are unlikely to be associated with treatment conditions, and therefore variance was measured by inter-quartile range and removed at 10 %. This filtering step yielded less than 10 features that fit the criterion for removal. Data was normalized by cumulative sum scaling and differential abundance analysis was conducted using metagenomeSeq with an FDR cut-off at q < 0.05. Microbiome Analyst was used to perform beta-diversity and relative abundance analysis. For quality control purposes, water and processed blank samples were sequenced and analyzed through the bioinformatics pipeline. Taxa identified as cyanobacteria or ‘unclassified’ to the phylum level were removed. Metadata and raw count data are provided in Supplemental Tables 4a,b.

### Statistical analysis

Data are represented as mean ± SD. Body weight, corticosterone response to acute stressors, cecal weight, and behavioral phenotyping data were analyzed by repeated measures analysis of variance and post-hoc analysis was conducted using Tukey’s correction for multiple comparisons. Given the nonparametric nature of microbiota data, indices of alpha diversity data was analyzed using Kruskal-Wallis test. Permutational multivariate analysis of variance was used to analyze effects of diet, sex and age. 16 S rRNA marker gene sequencing raw count data was filtered was set to include features where 20 % of its values contain a minimum of 4 counts. In addition, features that exhibit low variance across treatment conditions are unlikely to be associated with treatment conditions, and therefore variance was measured by inter-quartile range and removed at 10 %. Data was normalized by cumulative sum scaling and differential abundance analysis was conducted using Linear Discriminant Analysis effect size with an FDR cut-off at q < 0.05. Bar plots were visualized using GraphPad.

## Supplementary information

**Additional file 1**

**Additional file 2**

**Additional file 3**

**Additional file 4**

## Data Availability

No custom code or software was used for the analysis discussed in this manuscript. The associated data, including metadata and raw count data, generated and described in this published article is provided in Supplemental Tables 4a,b. Analyzed data is provided in Supplemental Tables 1, 2 and 3b.

## Abbreviations

Dox: Doxycycline
CON: Control
rRNA: ribosomal RNA
PCoA: principle coordinates analysis
ANOVA: analysis of variance
PERMANOVA: permutational analysis of variance
RM ANOVA: repeated measures analysis of variance
FDR: false discovery rate
